# Screening for and Verification of Novel Mutations Associated with Drug Resistance in the HIV Type 1subtype B^′^ in China

**DOI:** 10.1371/journal.pone.0047119

**Published:** 2012-11-08

**Authors:** Hanping Li, Qingmao Geng, Wei Guo, Daomin Zhuang, Lin Li, Yongjian Liu, Zuoyi Bao, Siyang Liu, Jingyun Li

**Affiliations:** Department of AIDS Research, State Key Laboratory of Pathogen and Biosecurity, Beijing Institute of Microbiology and Epidemiology, Beijing, China; National Institute of Health, United States of America

## Abstract

**Objective:**

Mutations associated with HIV drug resistance have been extensively characterized at the HIV-1 polymerase domain, but more studies have verified that mutations outside of the polymerase domain also results in resistance to antiviral drugs. In this study, mutations were identified in 354 patients experiencing antiretroviral therapy (ART) failure and in 97 naïve-therapy patients. Mutations whose impact on antiviral drugs was unknown were verified by phenotypic testing.

**Methods:**

Pol sequences of HIV subtype B^′^ obtained from patients experiencing ART failure and from naïve-therapy patients were analyzed for mutations distinct between two groups. Mutations that occurred at a significantly higher frequency in the ART failure than the naïve-therapy group were submitted to the Stanford HIV Drug Resistance Database (SHDB) to analyze the correlation between HIV mutations and drug resistance. For mutations whose impact on the antiviral drug response is unknown, the site-directed mutagenesis approach was applied to construct plasmids containing the screened mutations. 50% inhibitory concentration (IC_50_) to AZT, EFV and NVP was measured to determine the response of the genetically constructed viruses to antiviral drugs.

**Results:**

7 mutations at 6 positions of the RT region, D123E, V292I, K366R, T369A, T369V, A371V and I375V, occurred more frequently in the ART failure group than the naïve-therapy group. Phenotypic characterization of these HIV mutants revealed that constructed viruses with mutations A371V and T369V exhibited dual resistance to AZT and EFV respectively, whereas the other 5 mutations showed weak resistance. Although the impact of the other six mutations on response to NVP was minimal, mutation T369V could enhance resistance to NVP.

**Conclusions:**

This study demonstrated that mutations at the RT C-terminal in subtype B′ could result in resistance to RT inhibitors if the mutations occurred alone, but that some mutations could promote susceptibility to antiviral drugs.

## Introduction

Over 138 mutations in HIV-1 that are associated with drug resistance have been found since the first drug mutation was identified in 1989 [1]. 34 of these mutations at 15 positions associate with nucleoside reverse transcriptase inhibitors (NRTIs) and 19 mutations at 10 positions associate with non-nucleoside reverse transcriptase inhibitors (NNRTIs) at the reverse transcriptase (RT) region. There are 40 resistant mutations at 18 positions related with protease inhibitors (PIs) at the protease (PR) domain, and more than 30 mutations are associated with integrase inhibitors [2]. With the in-depth studies on drug resistance, resistant mutations related with the CCR5 inhibitor has also been identified and characterized [3]. Although the drug resistance mutations of HIV-1 usually occur at the polymerase domain, recent studies have verified that mutations at the RT C-terminal domains (connection and RNase H) also results in resistance to RT inhibitors [4]–[15].

Information about mutations in HIV-1 recorded in the HIV-1 drug resistance database is mostly derived from AIDS studies conducted with population overseas. Studies on the prevalence and occurrence of resistant strains in China remain relatively few as drug resistance started later and is probably concomitant with the increase popularity of free ART in 2003 [16]. In the subtype B [17], there are eight sites mutated at the p17 region and nine sites mutated at the V3 regions. The Thailand variant of subtype B is designated as subtype B′ and has spread for nearly thirty years [18]–[20]. Research have shown that the subtype B′ epidemics among infected paid blood donors (PBD) and heterosexuals in inland China most likely originated from a single founding subtype B′ strain that had been circulating among IDUs in Yunnan province [21]. This quickly became the most commonly transmitted HIV-1 subtype across the nation. Meanwhile, recombinant viruses that are subtype c and subtype C, CRF07_BCor CRF08_BC, have also became prevalent in China [22]–[23]. As one of the popular strains in China, the HIV-1 subtype B^′^ has been prevalent for a long time since it was introduced to China, and potentially experienced selective pressure under antiviral drugs since 2003. These reasons make it worthwhile to investigate whether novel mutations associated with drug resistance would exist in subtype B^′^. This paper aimed to screen and identify novel mutations associated with drug resistance in subtype B^′^ by identifying mutations at the pol region of HIV-1 that are present in the ART failure group but not in the naïve-therapy group.

## Results

### Patient characteristics

The plasma samples were collected from eight provinces, but most were derived from the central rural areas of China, such as Henan, Hebei and Shandong provinces (Table 1). A total of 451 sequences of HIV-1 subtype B′ were obtained, of which 97 were from the naïve-therapy patients and 354 were from the ART failure patients. The average age (IQR) was 37.9 years (23–49 years) and 47.3(32–69) respectively. Males predominated the study population, and the common transmission route was the PBD in the mid-1990s. The average viral load was 4.57lg (range from 3.13∼5.67) and 4.89lg (range from 3.18∼5.93) between the naïve and the ART failure population respectively, and the average CD4 count was 189 (range from 21∼343 cells/µl) and 207 cells/µl (range from 37∼410 cells/µl) respectively. The antiviral drugs were provided freely by the government, and the regimen of AZT/ddI/NVP was common(67.23%).

**Table 1:** Demographic characteristic of HIV-1 subtype B^′^ patients in study.

Characteristic	Patients Number(%)	
	Naïve(n = 97)	ART(n = 354)
**Age,median years(IQR)**	37.9(23–49)	47.3(31–69)
**Gender**		
Male	62(63.92)	249(70.34)
Female	35(36.08)	105(29.66)
**Median CD4+ T cell count, cells/µL(IQR)**	189(21–343)	207(37–410)
**Median viral load, RNA(lgcopies/ml)(IQR)**	4.57(3.13–5.67)	4.89(3.18–5.93)
**Risk of HIV infection**		
Heterosexual	16(16.49)	41(11.58)
Homosexual	5(5.15)	22(6.21)
IDU	11(11.34)	79(22.32)
PBD	61(62.89)	201(56.78)
Others/Unknown	4(4.12)	11(3.11)
**Provinces**		
Henan	43(44.33)	142(40.11)
Hebei	12(12.37)	67(18.93)
Shandong	19(19.59)	73(20.62)
Gansu	4(4.12)	14(3.95)
Guangdong	7(7.22)	21(5.93)
Guangxi Zhuang authority	9(9.28)	27(7.63)
Ningxia Hui authority	3(3.09)	10(2.82)
**ART regimens**		
AZT/ddI/NVP	/	238(67.23)
3TC/d4T/EFV	/	84(23.73)
AZT/3TC.NVP	/	32(9.04)

Note: AZT: zidovudine, d4T: stavudine, ddI: didanosine, 3TC: lamivudine, EFV: efavirenz, NVP: nevirapine.

### Screening for novel mutations associated with drug resistance in the ART patients

By comparing variations in the ancestor/consensus B sequences from naïve-therapy and ART groups, we detected no statistical difference in the variation at the PR domain between the naïve-therapy and the ART groups. A frequency of 16 mutations at the RT in the ART group was higher than in the naïve-therapy group (Table 2). The 9 mutations M41L, D67N, K70R, K103N, Y181C, M184V, T215Y, L283I and N348I resulted in resistance to NRTIs or NNRTIs, but the impact of 7 mutations at 6 positions (D123E, V292I, K366R, T369A, T369V, A371V and I375V) on antiviral drug response was unknown.

**Table 2:** 16 significant mutations between the ART and the naïve-therapy population.

Position	Codon of wild-type	Codon of mutation	Frequency (%)		?2	*p*	Impact on antiviral drugs response
			Naive	ART			
41	M	L	2.63	10.56	5.10	<0.05	R^a^
67	D	N	0	8.04	6.42	<0.05	R^a^
70	K	R	0	5.78	4.07	<0.05	R^a^
103	K	N	6.14	20.71	9.13	<0.01	R^a^
123	D	E	0	7.88	6.25	<0.05	U^b^
181	Y	C	2.63	13.43	7.90	<0.01	R^a^
184	M	V	0.88	10.19	8.29	<0.01	R^a^
215	T	Y	1.75	14.12	10.47	<0.01	R^a^
283	L	I	0	26.33	30.32	<0.01	R^a^
292	V	I	0	5.71	4.00	<0.05	U^b^
348	N	I	0	5.91	4.20	<0.05	R^a^
366	K	R	0	21.74	24.39	<0.01	U^b^
369	T	A	0	8.30	6.70	<0.01	U^b^
369	T	V	0	7.51	5.86	<0.05	U^b^
371	A	V	0	5.93	4.22	<0.05	U^b^
375	I	V	0	7.20	5.54	<0.05	U^b^

Note:

a R indicated mutations that could confer resistance to antiviral drugs, and

b U indicated mutations which confers an unknown response to antiviral drugs.

Among the 7 screened mutations, 2 mutations D123E and V292I located at the polymerase domain and 5 (K366R, T369A, T369V, A371V and I375V) at the RT connection domain were further analyzed on the relationship between the mutations and drug resistance in SHDB. The mutation D123E accounted for 39% of the 12 classes of mutations at position 123. The predominant mutations were V292I and K366R at codons 292 and 366 respectively, whose frequencies were 99% among all mutations at the corresponding position. Five classes of mutations were observed at codon 369, with T369A (61%) and T369V (34%) as most common. As the popular mutation pattern, mutations A371V and I375V were observed in 94% of ART patients. These results suggest that certain mutations that occur at a higher frequency than other mutations may have been conferred a selective advantage over other mutations at the corresponding codon position.

### Assaying for the susceptibility of the mutant viruses to antiviral drugs

**Figure 1:**
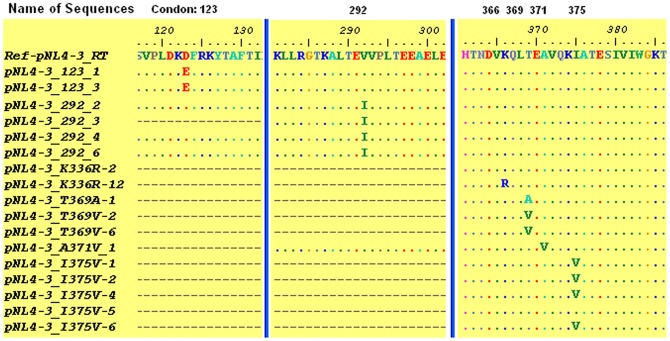
Sequencing results of the constructed plasmid pNL4-3 with mutations. All sequences were obtained from a single colony, and sequencing results from the supernatant/harvest virions matched the sequence from the single colony, suggesting that the mutations were not altered during transfection and infection.

**Site-directed mutagenesis and transfection** The mutations were introduced into the plasmid pNL4-3 by site-directed mutagenesis. The constructed plasmids were transformed in *E.coli DH5α.* Single colonies were sequenced to determine whether the plasmids containing mutations were constructed successfully (Figure 1).

### Generation of viral particles

The cell supernatants were collected after transfection of the mutant plasmids into 293T cells, and then the supernatant/harvested virions were used to infect MT2 cells. The presence of a massive syncytia in MT-2 cells indicated that the virus particles obtained through transfection were infectious by CPE. Further sequencing of the virus particles verified that each virus particle contained the corresponding mutation at the proper position, and the results of sequencing were shown in Figure 1.

### Of three antiviral drugs for the IC50 determination of constructed viruses

The TCID50 of each constructed mutant virus was more than 10^3^, suggesting sufficient viral titer for subsequent experiments. The impact of three antiviral drugs on the mutant viruses was measured by change fold in IC_50_ between the mutant viruses and the pNL4-3 wild-type virus(pNL4-3_-wild_).

**Figure 2:**
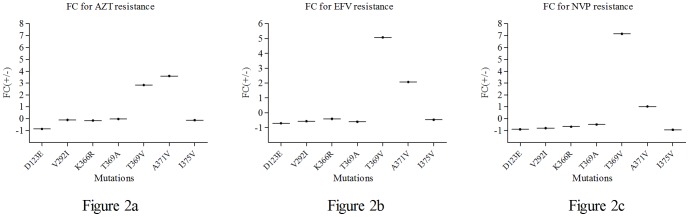
Relationship between viral replication and antiviral drugs at various concentration. The effect of the drugs AZT, EFV and NVP on mutant viruses pNL4-3_T369A_ and pNL4-3_T369V_ was illustrated. This map was drawn using Graphpad Prism ® software, and the abscissa represents the different drug concentrations (lgnM) and the vertical axis represents the inhibition ratio of drugs on the virus. **Figure 2a** Relationship between viral replication and AZT concentration. **Figure 2b** Relationship between viral replication and EFV concentration. **Figure 2b** Relationship between viral replication and NVP concentration.

The IC_50_ of the mutant viruses to three antiviral drugs were listed in Table 3. The D234E mutation does not confer a change in viral resistance to AZT as the IC_50_ for the mutant virus pNL4-3 _D123E_ was not significantly different from that of pNL4-3_-wild_. Compared with the wild type virus pNL4-3_wild_, the mutant viruses pNL4-3_T369V_ and pNL4-3_A371V_ exhibited a low resistance to AZT, with their IC_50_ increased by 2.83 and 3.60 fold, respectively (Figure 2a,3a). IC_50_ of as the mutant viruses pNL4-3_T369A_, pNL4-3_V292I_, pNL4-3_I375V_ and pNL4-3_K366R_ were as follows: 3.15 nM, 11.77 nM, 14.51 nM and 17.29 nM, but IC_50_ of pNL4-3_wild_ was 112.50 nM. These observations suggest that the 4 mutations listed could promote fitness under conditions where the wild type virus would have been susceptible.

**Figure 3:**
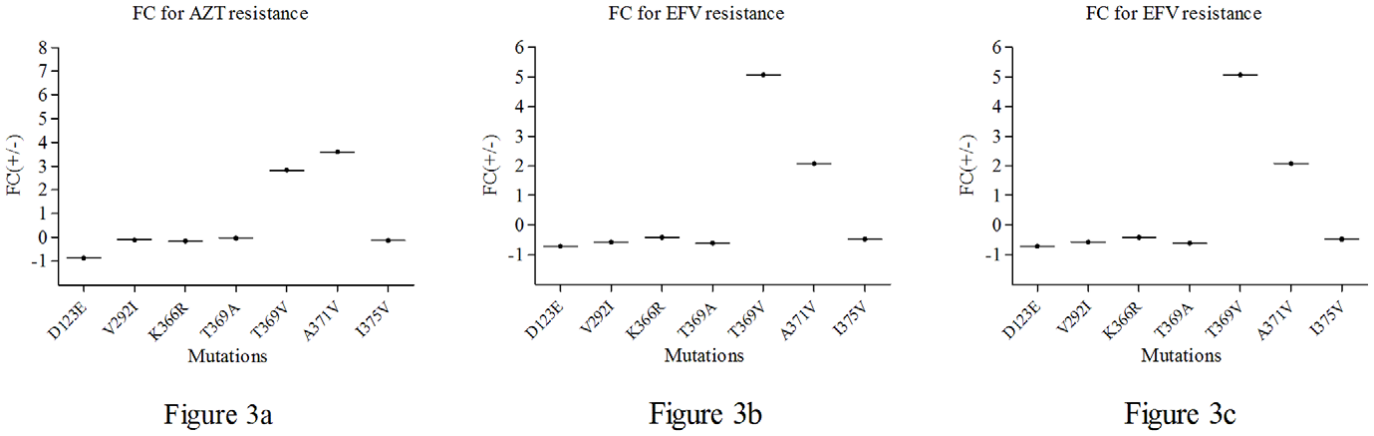
Fold change in the response of mutant viruses to antiviral drugs. When IC_50_ of the mutant virus was greater than that of the wild-type virus, the fold change(FC) was calculated as IC_50_ of mutant virus/wild-type virus and then indicated as positive (+); When IC_50_ of the mutant virus was less than that of the wild-type, the fold change was indicated as negative (−). Figure 3a Fold change in the response of mutant viruses to AZT. Figure 3b Fold change in the response of mutant viruses to EFV. Figure 3c Fold change in the response of mutant viruses to NVP.

**Table 3:** IC_50_ (nM) of constructed viruses for three antiviral drugs.

Reconstruction viruses	IC_50_ of antiviral drugs		
	AZT	EFV	NVP
pNL4.3_D123E_	97.35±21.97	6.94±2.94	491.28±215.59
pNL4.3_V292I_	11.77±6.35	5.56±4.10	434.90±240.58
pNL4.3_K366R_	17.29±9.48	4.13±2.27	363.23±173.24
pNL4.3_T369A_	3.15±1.97	5.80±3.81	268.85±88.68
pNL4.3_T369V_	318.10±109.24	49.39±30.37	3899.00±2064.60
pNL4.3_A371V_	404.80±294.32	20.19±12.35	562.95±223.32
pNL4.3_I375V_	14.51±9.17	4.53±4.50	515.25±192.28
pNL4.3_wild_	112.50±68.25	9.72±2.07	546.13±225.09

* The data are the means of four experiments±standard deviations.

The mutant viruses pNL4-3_T369V_ and pNL4-3_A371V_ could enhance resistance to EFV given that their FC increased by 5.08 and 2.08 fold respectively (Figure 2b,3b). However, the other five mutant viruses pNL4-3_D123E_, pNL4-3_V292I_, pNL4-3_K366R_, pNL4-3_T369A_ and pNL4-3_I375V_ exhibited enhanced susceptibility to EFV for 1∼2 fold compared with pNL4-3_wild_(9.72 nM). The IC_50_ for the mutant viruses were 6.94 nM, 5.56 nM, 4.13 nM, 5.80 nM and 4.53 nM, respectively.

With NVP, the virus pNL4-3_T369V_ exhibited more fitness in viral replication compared with control. Its IC_50_ attained 3899.00 nM but the IC_50_ of pNL4-3_wild_ was only 546.13 nM, and the FC was 7.14-fold (Figure 2c,3c). No considerable difference was seen in the NVP response of the 6 other mutant viruses compared with the wild type virus.

## Discussion

HIV-1 RT is an asymmetric dimer composed of a 51 kD subunit (p51) and a 66 kD subunit (p66). The subunit p66 which contains the catalytic activity consists of the polymerase domain (1–315 aa), connecting domain (316–437 aa) and RNase H domain (RNase H, 438–560 aa). Polymerase domain could be subdivided into three sub-domains of p51 finger domain (fingers), palm domain (palm) and thumb domains (thumb). Interestingly, the known resistance mutations related with NRTI are mainly located in the palm domain and RT fingers domain which help bind the RNA template onto the catalytic sites. The resistant mutations related with NNRTI are mainly located in the palm domain. In studies on HIV-1 drug resistance, many novel mutations related with resistance are found on the RT catalytic activity domain, especially at the connection domain. Nikolenko *et al* reported in 2007 that such mutations as E312Q, G335C/D, N348I, A360I/V, V365I and A376S at the connection domain could confer resistance to AZT to a certain extent [4]–[15]. Mutation N348I was verified by Yap *et al* at the end of 2007 to result in low resistance to AZT, with its degree of AZT resistance increasing with the number of TAMs [24]. Brehm *et al* also reported in 2007 that mutation A371V at the connection domain could enhance the resistance level to AZT along with TAMs [25]. These studies suggest that mutations related with drug resistance usually enhance the degree of resistance in combination with TAMs. Latter studies confirmed these observations [26]–[28].

Comparing 354 sequences obtained from ART patients with 97-naïve sequences, 7 mutations at 6 positions in RT domain were screened and their variation patterns and frequencies were investigated using SHDB. The frequencies of these mutations in ART patients whose regimens contained some antiviral drugs were significantly higher than in the naïve-therapy patients. This suggests that the occurrence of these mutations is in close association with the use of antiviral drugs.

Two mutations (D123E and V292I) identified in this study are located at the DNA polymerase domain and the other 5 (K366R, T369A, T369V, A371V and I375V) at the connection domain. Phenotypic tests verified that the mutations A371V and T369V could enhance resistance to AZT and EFV, whereas the other 5 mutations could enhance the replication fitness of viruses relative to pNL4-3_wild_. The susceptibility test for NVP showed that the resistant level of virus pNL4-3_T369V_ was enhanced whereas the replication fitness of the other 6 mutant viruses was superior to the virus pNL4-3_wild_. Interestingly, mutation at position 369 of RT for variation T369A could enhance the susceptibility of virus to AZT and EFV, but the variation T369V resulted in resistance to AZT, EFV and NVP, a finding not reported previously. Although the mutation at 369 position appeared inconsequential, namely T369A at the first codon (ACA→GCA) and T369V at the first two codon(ACA→GTA), the impact on drug susceptibility was great. The discrepancy of IC_50_ for AZT between viruses pNL4-3_T369A_ and pNL4-3_T369V_ was 101-fold, and variation T369A promoted the replication fitness of virus but T369V enhanced the resistance level of virus to antiviral drugs. The impact of 2 mutations mentioned above on EFV was similar with AZT, and the discrepancy was 7-fold. The impact of T369V on NVP was significant, for the fold change was 5.08 fold relative to wild type virus pNL4-3_wild_. These results suggest that mutations at the 369 position were vital to the replication fitness of the virus. In addition, mutations K336R and I375V at the connection domain could promote the replication fitness of virus pNL4-3 under AZT and EFV exposure, relative to pNL4-3_wild_.

The resistance-associated mutations were identified primarily on non-B subtypes in recent years for there was an increasing prevalence of non-B subtype HIV-1 infections in the world. Although the mutations associated with resistance in subtype B has been characterized in detail, the impact of the resistance-associated mutations on non-B subtypes remains unknown. This is because the interpretation of genotypic resistance tests depends on the commercial kits or the web-based HIV-1 resistance interpretation tools that were established according to the background sequences of HIV-1 subtype B. Given the difference between the sequences of subtype B and non-B subtypes [29]–[30], the present tools used in genotypic tests are unable to provide the proper interpretation for the non-B subtypes. Mutations differ across various subtypes according to previous reports [31]–[32]. The prevalence of TAMs-I and TAMs-II, for example, was different between CRF02_AG and subtype F, and some differences in the resistance-associated mutations have been found among patients infected with B, C, F, and CRF02_AG subtypes. Mutations at the protease regions such as 20MRI, 36I, and 89IMT are more prevalent among non-B subtypes, but mutations 84V, 10FR, 63P, 71LTV and 77I are common in subtype B [33]. The systemic review on the distinct resistant mutations among HIV-1 was conducted by Martinez-Cajas JL and collaborators in 2009 [34], which indicated formally that the differences among known resistant-associated mutations between the subtype B and the non-B subtypes are marginal because the differences are primarily minor mutations. Although frequencies of the screened mutations in other non-B subtypes in this study was not well-described, the presence of specific polymorphisms at the C-terminal domains was characterized in a study in Southern Brazil [35]. The review mentioned that the compensatory mutation D488E occurs more frequently in subtype C than in subtype B, while the inverse is true for mutation Q547Q. Mutations T369V/I and A371V emerged in the drug-naïve patients, but the frequencies were relatively low and there was a significant difference between the subtype B and the subtype C. The mutation A371V is considered a polymorphism of CRF01_AE in the C-terminal half of RT, which did not confer resistance by itself but conferred significant resistance to NRTIs with TAMs, especially combined with TAM-II [36]. A limitation of this study is that the frequencies of screened mutations among the predominantly circulating subtypes such as CRF_BC and CRF_01AE were not described in detail, and so it remains undetermined whether our results were drug mutations unique to particular subtypes and could confer a difference in clinical response to ART. We inferred from previous findings that the impact of mutations T369V and A371V on RT inhibitors is not significant if occurring alone, but the resistance level would be enhanced if the mutations occur in combination with TAMs. Our findings showed that the resistance level was enhanced by 2.08∼7.14 FCs to NNRTIs when the mutations T369V or A371V occurred alone. We are currently investigating the impact of these mutations in tandem with TAMS on susceptibility to RT inhibitors. We were limited by the difficulty in recruiting naïve-therapy individuals undergoing free ART, and thus, the sequences of the naïve-therapy individuals were not enough for a statistical comparison among experimental groups. A possible bias exists in our analysis of mutations between the ART population and the naïve-therapy population, but we considered the chance of this to be slim and it would not impact our experimental results. The results in this study not only supplemented information about HIV drug resistance, but also improved genotypic resistance testing, which suggests that current resistance assays should not be limited to known mutations and usual drug targets. As we have observed in this study, mutations outside of the catalytic activity sites similarly played an important role in the occurrence and development of drug resistance. There is usually insufficient understanding about phenotypes resulting from a single mutation, as the interaction among mutations is complex and their effects on antiviral drug response is usually counteracted each other. In addition, it is possible that there exists more novel mutations in the HIV-1 genome whose impact on drug resistance remains unknown due to limitations in genotypic resistance tests. With the mutual complementarity between the genotype and the phenotype in resistance assays, more mutations associated with resistance could be found and verified.

## Materials and Methods

### Ethics Statement

Written informed consent was obtained for every participant before the anticoagulated venous blood was collected by trained medical staffs. The study was revised and approved by the Ethics Committee of Institutional Review Board of Academy of Military Medical Sciences.

### Sequences of HIV-1 pol gene and their origins

Sequences of HIV-1 pol gene were obtained through the surveillance of HIV-1 drug resistance in such centers for Disease Control and Prevention of Henan, Hebei, Shandong, Gansu, Beijing,Guangdong, Guangxi Zhuang authority and Ningxia Hui authority from 2004 to 2010. The amplification segment of pol gene was 1977 bp, which comprised the entire gene of PR (codons 1–99) and RT(codons 1–560), and sequence quality was assessed using the WHO sequence quality-assessment tool. The subtypes of obtained sequences were verified by the Ref 08-pol downloaded from the website http://www.hiv.lanl.gov. Among the obtained pol sequences, a total of 451 sequences were that of subtype B^′^, including 97 from the naïve-therapy group and 354 from the ART failure. The percentage of ART patients undergoing specific drug regimens including AZT(Zidovudine)/ddI(Didanosine)/NVP(Nevirapine), 3TC(Lamivudine)/d4T(Stavudine)/EFV(Efavirenz) and AZT/3TC/NVP were 67.23%, 23.73%, 9.04%, respectively, and the duration of therapy was more than 12 months.

### Screening of novel mutations

Mutations whose frequencies were significantly higher in the ART than the naïve-therapy population were identified. The potential impact of the screened mutations on antiviral drugs response was assessed by submitting the sequences to SHDB. Mutations whose impact on antiviral drugs was unknown were subjected to phenotypic test.

### Plasmid construction and transfection

The plasmids containing the identified screened mutations were constructed by site-directed mutagenesis on the plasmid pNL4-3. The constructed plasmids were transfected to 293 T cells to obtain the viruses to be used for infection. The strategy used to construct plasmid for phenotypic resistance analysis was depictedas following. Site-directed mutagenesis on the plasmid pNL4-3 was difficult because of its large size (about 15 Kb), so the RT genes (position 1435–3514, 3231 to 6019) were amplified from the plasmid pNL4-3 and ligated into the vector PMD 18T(TaKaRa) respectively. Site-directed mutagenesis was then conducted with Phusion™ Site-directed Mutagenesis Kit(New England Biolabs) and the following mutations were introduced: D123E, V292I, K366R, T369A, T369V, A371V and I375V. The primers used for amplification and site-directed mutagenesis were listed in Table 4.The targeted segments digested by the *AgeI* and *SphI* were subcloned into the plasmid pNL4-3, then the constructed plasmids were transfected into 293 T cells(at a density of 4×10^5^ cells/ml) using Lipofectamine™ 2000 (Invitrogen) to obtain the virus. Transfection was conducted according to manufacturer's instructions (Lipofectamine™ 2000, Invitrogen). Forty-eight hours after transfection at 37°C incubator, culture supernatants were harvested and stored at −80°C freezer until use.

**Table 4:** Primers of the site-directed mutagenesis.

Primer	Sequence (5′ - 3′)	Location (pNL4-3)
MAW-F26^a^	TGGAAATGTGGAAAGGAAGGAC	2029–2050
DO2-R1^a^	CTGTCCCTGTAATAAACCCGAAA	4918–4896
PLA-7^a^	CTTTGGATGGGTTATGAACT	3231–3250
Vif-3^a^	TCGCTGTCTTCGCTTCTTCCTGCCAT	5994–6019
SEQ-R3^b^	CTTCTGTATATCATTGACAGTCCAGCT	3300–3326
SEQ-F4^b^	CCTAGTATAAACAATGAGACAC	2946–2967
SEQ-R6^b^	TAAAATCACTAGCCATTGCTCTCC	4285–4308
D123E_F^c^	P-TCCCTTAGATAAAGAATTCAGGAAGTATACTG*	2903–2935
D123E_R^c^	P-ACTGAAAAATATGCATCGCCC	2902–2882
V292I_F^c^	P-CCAAAGCACTAACAGAAATAGTACCACTAACAG	3406–3438
V292I_R^c^	P-TTCCCCTAAGAAGTTTACATAATTGCCTTAC	3405–3375
K366R_F^c^	P-CACTAATGATGTGAGACAATTAACAGAGGCAG	3632–3663
K366R_R^c^	P-TGGGCACCCTTCATTCTTGCATAT	3631–3608
T369A_F^c^	P-GATGTGAAACAATTAGCAGAGGCAGTACAAA	3639–3669
T369A_R^c^	P-ATTAGTGTGGGCACCCTTCATTCTT	3638–3614
T369V_F^c^	P-GATGTGAAACAATTAGTAGAGGCAGTACAAA	3639–3669
T369V_R^c^	P-ATTAGTGTGGGCACCCTTCATTC	3638–3616
A371V_F^c^	P-GAAACAATTAACAGAGGTAGTACAAAAAATAGCC	3644–3677
A371V_R^c^	P-ACATCATTAGTGTGGGCACCCTT	3643–3621
I375V_F^c^	P-GGCAGTACAAAAAGTAGCCACAGAAAG	3659–3685
I375V_R^c^	P-TCTGTTAATTGTTTCACATCATTAGTGTGG	3658–3629

Note:

a The primers were used to amplify the targeted fragments from the plasmid pNL4-3.

b SEQ-R3,SEQ-F4 and SEQ-R6 were sequencing primers.

c F and R represented the forward and the reverse primer at the site-directed mutagenesis, and P represented the phosphorylation at the 5′ termination. The bases underlined were mutant bases.

### The drug susceptibility assays of constructed mutant viruses

The replication fitness of the constructed mutant viruses was assessed through the typical cytopathic effect(CPE) at various drug concentration. The culture supernatants stored at −80°C freezer were diluted to 20 µl according to the multiplicity of infection(MOI, 0.003366TCID50) and plated into the 96-well plate containing antiviral drugs(AZT, EFV and NVP), then 293T cells at the final concentration of 7×10^4^ cells/ml was added into the corresponding well and the 96-well plate was transferred to 37°C CO_2_ (5%) incubator. 50 µl RPMI-1640 medium (10%FBS,Prep/Strep) was added to the corresponding well after 3 days and the inhibition ratio was calculated according to the CPE at different drug concentration observed after 5 days. Inhibition curves between the mutant viruses and antiviral drugs were drawn using Graphpad Prism®, and the IC_50_ of each virus to AZT, EFV and NVP was calculated according to inhibition ratio of virus with mutations at different drug concentrations.

### Statistical analysis

*Chi-square* test was used to calculate the difference of mutations between the ART and the naïve-therapy population. All analyses were performed using a two-tailed *P* values and *P* values less than 0.05 were considered statistically significant.
